# Characterization of transcriptome dynamics during watermelon fruit development: sequencing, assembly, annotation and gene expression profiles

**DOI:** 10.1186/1471-2164-12-454

**Published:** 2011-09-21

**Authors:** Shaogui Guo, Jingan Liu, Yi Zheng, Mingyun Huang, Haiying Zhang, Guoyi Gong, Hongju He, Yi Ren, Silin Zhong, Zhangjun Fei, Yong Xu

**Affiliations:** 1 National Engineering Research Center for Vegetables, Beijing Academy of Agriculture and Forestry Sciences, Beijing 100097, China; 2 Boyce Thompson Institute for Plant Research, Cornell University, Ithaca, NY 14853, USA; 3 USDA Robert W. Holley Center for Agriculture and Health, Tower Road, Ithaca, NY 14853, USA

## Abstract

**Background:**

Cultivated watermelon [*Citrullus lanatus *(Thunb.) Matsum. & Nakai var. *lanatus*] is an important agriculture crop world-wide. The fruit of watermelon undergoes distinct stages of development with dramatic changes in its size, color, sweetness, texture and aroma. In order to better understand the genetic and molecular basis of these changes and significantly expand the watermelon transcript catalog, we have selected four critical stages of watermelon fruit development and used Roche/454 next-generation sequencing technology to generate a large expressed sequence tag (EST) dataset and a comprehensive transcriptome profile for watermelon fruit flesh tissues.

**Results:**

We performed half Roche/454 GS-FLX run for each of the four watermelon fruit developmental stages (immature white, white-pink flesh, red flesh and over-ripe) and obtained 577,023 high quality ESTs with an average length of 302.8 bp. *De novo *assembly of these ESTs together with 11,786 watermelon ESTs collected from GenBank produced 75,068 unigenes with a total length of approximately 31.8 Mb. Overall 54.9% of the unigenes showed significant similarities to known sequences in GenBank non-redundant (nr) protein database and around two-thirds of them matched proteins of cucumber, the most closely-related species with a sequenced genome. The unigenes were further assigned with gene ontology (GO) terms and mapped to biochemical pathways. More than 5,000 SSRs were identified from the EST collection. Furthermore we carried out digital gene expression analysis of these ESTs and identified 3,023 genes that were differentially expressed during watermelon fruit development and ripening, which provided novel insights into watermelon fruit biology and a comprehensive resource of candidate genes for future functional analysis. We then generated profiles of several interesting metabolites that are important to fruit quality including pigmentation and sweetness. Integrative analysis of metabolite and digital gene expression profiles helped elucidating molecular mechanisms governing these important quality-related traits during watermelon fruit development.

**Conclusion:**

We have generated a large collection of watermelon ESTs, which represents a significant expansion of the current transcript catalog of watermelon and a valuable resource for future studies on the genomics of watermelon and other closely-related species. Digital expression analysis of this EST collection allowed us to identify a large set of genes that were differentially expressed during watermelon fruit development and ripening, which provide a rich source of candidates for future functional analysis and represent a valuable increase in our knowledge base of watermelon fruit biology.

## Background

Watermelon [*Citrullus lanatus *(Thunb.) Matsum. & Nakai var. *lanatus*] belongs to the *Cucurbitaceae *family which includes several other important vegetable crops such as melon, cucumber, squash and pumpkin. It produces large edible fruits that serve as an important component in human diets throughout the world [1] and its farming accounts for ~7% of the world's total area devoted to vegetable production according to FAO statistics [2]. Its production in the U.S. alone reached 4 billion pounds in 2010 with a net market value of half billion U.S. dollars. The quality of watermelon fruits consists of many factors including fruit shape and size, rind thickness and color, flesh texture and color, aroma, flavor, sugar content, carotenoid and flavonoid composition, and nutrient composition [3]. During the development and ripening process, watermelon fruits undergo many biochemical and physiological changes including size expansion, fruit softening, and accumulation of sugars, pigments, and flavor and aromatic volatiles [4,5]. Most of these traits are controlled by multiple QTLs and pose a significant challenge to traditional breeding [6,7].

Currently genomics and functional genomics resources of watermelon that are publicly available are very limited. This lack of extensive genomics and functional genomics resources, combined with the narrow genetic diversity among watermelon cultivars, is one of the major limiting factors in watermelon research and breeding. However, this situation will soon be changed due to the recent advent of next-generation sequencing (NGS) technologies such as Roche/454 and Illumina/Solexa sequencing platforms. The extremely high throughput and relatively low cost of these sequencing technologies have offered unique opportunities to study genomics and functional genomics in non-model organisms. Using the NGS technologies, currently the genome of watermelon, which has an estimated size of 425 Mb [8], is being sequenced by the International Watermelon Genomics Initiative. The genome sequencing of cucumber, a closely-related cucurbit species, was completed [9], and the genome of melon, another closely-related cucurbit species, is being sequenced under the Spanish Genomics Initiative (MELONOMICS). Complementary to whole genome sequencing, which still requires huge effort and investment, large-scale transcriptome sequencing has proved to be efficient and cost-effective for gene discovery and gene function and expression analysis. Recently, several large expressed sequence tag (EST) datasets have been generated in cucurbit species. These include approximately 1.2 million, 350,000 and 500,000 ESTs generated from melon [10], cucumber [11] and *Cucurbita pepo *[12], respectively, using the Roche/454 sequencing technologies, and an additional ~127,000 ESTs generated from melon using the traditional Sanger sequencing approach [13]. However, currently only around 12,000 watermelon ESTs are available in GenBank. Thus it's very important to expand the transcript catalog of watermelon in order to facilitate gene discovery, functional analysis, molecular breeding, and comparative genomics analysis of watermelon and its closely-related species.

Fruit is a major component of the human diet contributing a large portion of vitamins, minerals, antioxidants and fiber. Fruit development and ripening is a complex process influenced by numerous factors including light, hormones, temperature and genotype. Ripening associated events are brought about by developmentally and physiologically regulated changes in gene expression which ultimately lead to alterations in color, texture, flavor, and aroma of fruit. Fruit can be physiologically classified as climacteric or non-climacteric depending on the presence or absence of a burst in respiration at the onset of ripening [14]. Tomato, a climacteric fruit, has served as the model system for fruit development and ripening and attracted extensive studies to understand molecular mechanisms of the development and ripening of its fleshy fruits [15]. However, very little information is currently available at the molecular level regarding fruit development and ripening of watermelon, a non-climacteric and economically important fruit crop. To date, there is only one report describing expression profiles of a very small set of genes (832) during watermelon fruit development through microarray and qRT-PCR analysis [3].

In order to significantly expand the transcript catalog of watermelon and gain more insights into the molecular basis of watermelon fruit development, we performed large-scale transcriptome sequencing of watermelon fruits using the Roch/454 massively parallel pyrosequencing technology. A total of 577,023 ESTs were obtained and assembled into 75,068 unigenes which were further extensively annotated. Digital expression analysis of these ESTs identified more than 3,000 unigenes that were differentially expressed during watermelon fruit development. This information, coupled with profiles of several interesting metabolites that are important to fruit quality, provided novel insights into the biology of watermelon fruit development and ripening process.

## Results and discussion

### Sequencing and assembly of watermelon fruit transcriptome

**Figure 1:**
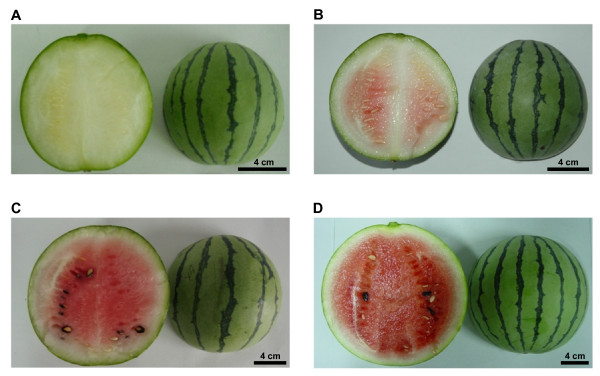
**Fruits of watermelon cultivar 97103 at immature white - 10 DAP (A), white-pink flesh - 18 DAP (B), red flesh - 26 DAP (C) and over-ripe - 34 DAP (D) stages**. DAP: days after pollination.

Watermelon cultivar 97103 was used in the present study. It is a typical East Asian cultivar that produces round shape, medium size, thin rind, and green skin fruits with light red flesh. Four critical stages of fruit development, immature white (10 days after pollination (DAP)), white-pink flesh (18 DAP), red flesh (26 DAP) and over-ripe (34 DAP), were examined (Figure 1). Fruits at the immature white stage undergo rapid cell division and expansion leading to significant increase of fruit size and weight. At this stage, there is no distinguishable difference between the fruit inner peel and the flesh tissue in term of texture and color. Its soluble solid content (SSC) is also considerably lower than that of the mature fruit (Additional file 1). At the white-pink flesh stage, the fruit continue to expand without much increase in SSC, but the fruit flesh begins to turn pink and it starts to lose its firmness (Additional file 1). After reaching the red flesh stage, the fruit is fully mature and its flesh becomes light red, much crispier and sweeter. The changes of texture and taste are also associated with a rapid increase of SSC (Additional file 1). At the over-ripe stage, the fruit is now over-matured and the flesh turns bright red with accumulation of volatile compounds that gives watermelon its distinct aroma and flavor (Figure 1).

**Figure 2:**
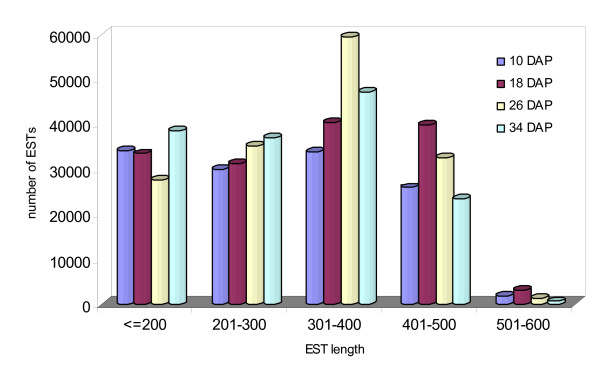
**Length distribution of watermelon ESTs**.

To characterize watermelon transcriptome and generate expression profiles for fruit development, we used the Roche/454 GS-FLX (Titanium) pyrosequencing technology to sequence cDNA samples from the four aforementioned fruit developmental stages. A half run was performed for each of the four fruit samples and approximately 800,000 raw reads were obtained. After trimming low quality regions and removing short (< 100 bp) and contaminated reads, we obtained a total of 577,023 high quality ESTs with an average length of 302.8 bp and total length of 174.7 Mb (Table 1). The length distribution of these high quality Roche/454 ESTs in each sample is shown in Figure 2. Over 75% of these ESTs fell between 200 to 500 bp in length.

**Table 1:** Statistics of watermelon Roche/454 ESTs

	10 DAP	18 DAP	26 DAP	34 DAP	Total
**No. of reads**	125,724	148,143	156,299	146,857	577,023
**Average length (bp)**	293.8	314	313.7	287.5	302.8
**Total bases (bp)**	36,939,267	46,511,849	49,031,126	42,224,795	174,707,037
**No. of reads in contigs**	115,482	137,955	150,548	142,862	546,847
**No. of reads in singletons**	10,242	10,188	5,751	3,995	30,176

The Roche/454 ESTs generated in the present study, together with 11,786 watermelon Sanger ESTs collected from GenBank dbEST database, were *de novo *assembled into 75,068 unigenes with an average length of 424 bp. The unigenes included 43,544 contigs with an average length of 540.3 bp and 31,524 singletons with an average length of 263.4 bp. The assembled transcriptome is approximately 31.8 Mb in size. Table 2 lists the distribution of the number of EST members in watermelon unigenes. Around 10% of the unigenes (7,568 transcripts) have more than 10 EST members and they contain ~71% of the total EST reads. The EST and unigene sequences generated in this study are freely available at the Cucurbit Genomics Database [16].

**Table 2:** Distribution of number of ESTs in watermelon unigenes

No. EST members	No. unigenes	No. ESTs in unigene
1	31,524	31,524
2	13,925	27,850
3	7,413	22,239
4	4,561	18,244
5	3,159	15,795
6	2,254	13,524
7	1,592	11,144
8	1,277	10,216
9	994	8,946
10	801	8,010
11-30	5,020	85,919
31-50	1,071	41,529
51-70	457	27,022
71-90	243	19,502
91-110	143	14,353
> 110	634	229,838

Our assembly included approximately 8,000 ESTs generated from a fruit normalized and subtracted library reported in Levi et al. [17]. In the assembly, these ESTs were distributed in 4,616 unigenes, among which 962 (20.8%) were not captured by our 454 deep transcriptome sequencing. This indicated that although the 454 deep sequencing generated a large number of novel unigenes (more than 70,000), sequencing the transcriptome to higher depth is required to discover more rare genes.

### Functional annotation, comparative genomics and pathway analysis

To annotate the watermelon transcriptome, we first compared unigene sequences against the NCBI non-redundant (nr) protein database using the BLASTX program. The analysis revealed that 41,245 (54.9%), 20,648 (27.5%), and 4,493 (5.9%) unigenes had matches to known protein sequences with E-values < 1e-5, 1e-20, and 1e-50, respectively (Table 3). As expected, much higher proportion of contigs had sequence matches than singletons (67.9% vs 37.1% at E-value < 1e-5) due to the much shorter average length of singletons which are less likely to contain coding regions. We also compared watermelon unigenes against UniProt (SwissProt and TrEMBL) protein databases. Details of these sequence comparisons are provided at the Cucurbit Genomics Database [16].

**Table 3:** Statistics of watermelon unigenes with sequence matches against public protein databases

Database	E value	Contig	Singleton	Total
NCBI nr	1e-5	29,550 (67.9%)	11,695 (37.1%)	41,245 (54.9%)
	1e-20	16,496 (37.9%)	4,152 (13.2%)	20,648 (27.5%)
	1e-50	3,933 (9%)	460 (1.5%)	4,393 (5.9%)

cucumber protein	1e-5	33,708 (77.4%)	15,716 (49.9%)	49,424 (65.8%)
	1e-20	23,692 (54.4%)	7,672 (24.3%)	31,364 (41.8%)
	1e-50	7,969 (18.3)	1,241 (3.9%)	9,210 (12.3%)

Arabidopsis protein	1e-5	29,409 (67.5%)	11,925 (37.8%)	41,334 (55.1%)
	1e-20	17,433 (40%)	4,501 (14.3%)	21,934 (29.2%)
	1e-50	4,516 (10.4%)	473 (1.5%)	4,989 (6.6%)

rice protein	1e-5	28,107 (64.5%)	10,843 (34.4%)	38,950 (51.9%)
	1e-20	15,769 (36.2%)	3,833 (12.2%)	19,602 (26.1%)
	1e-50	3,944 (9.1%)	336 (1.1%)	4,280 (5.7%)

Note: percentages of contigs, singletons and total unigenes were calculated from total numbers of contigs (43,544), singletons (31,524) and unigenes (75,068), respectively.

**Figure 3:**
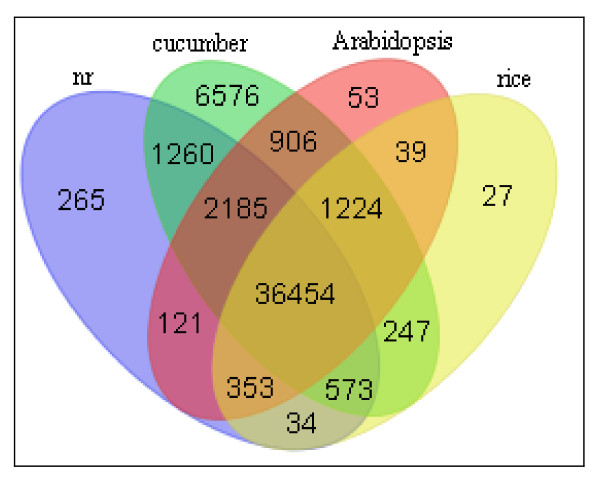
**Venn diagram of number of watermelon unigenes with sequence matches against GenBank nr, cucumber, Arabidopsis, and rice protein databases**. Numbers of sequence matches at an E-value cutoff of 1e-5 are shown.

We then compared watermelon unigenes against protein database of cucumber, the closely related cucurbit species with a sequenced genome, and Arabidopsis and rice, model systems for dicot and monocot plants, respectively. At an E-value < 1e-5, approximately 66% of watermelon unigenes had matches in cucumber protein database, while 55% and 52% of watermelon unigenes in Arabidopsis and rice protein databases, respectively (Table 3). Of watermelon unigenes that had matches in at least one of the three databases, the majority (~80%) had sequence matches in all three protein databases (Figure 3). As shown in Figure 3, a significant number of watermelon unigenes (~9,000) that did not have matches against nr did have matches against cucumber proteins. This is not unexpected since currently cucumber proteins have yet to be archived in the nr database, and watermelon and cucumber are closely-related *Cucurbitaceae *species.

We further compared watermelon unigenes against pfam domain database [18]. A total of 11,454 watermelon unigenes contained at least one pfam domain and 1,475 distinct pfam domains were represented by the unigenes. The most abundant pfam domains found in the collection of watermelon unigenes were PF01439 (metallothionein; 343 unigenes), PF00179 (ubiquitin-conjugating enzyme; 278), PF01200 (ribosomal protein S28e; 223), PF06522 (B12D protein; 186), and PF00025 (ADP-ribosylation factor family; 183). We also found that 465 unigenes contained transcription factor domains. The most represented pfam transcription factor domains were PF00319 (SRF-type transcription factor; 50), PF00249 (Myb-like DNA-binding domain; 42), PF00847 (AP2 domain; 37), PF00046 (homeobox domain; 27), and PF01486 (K-box region; 27).

**Figure 4:**
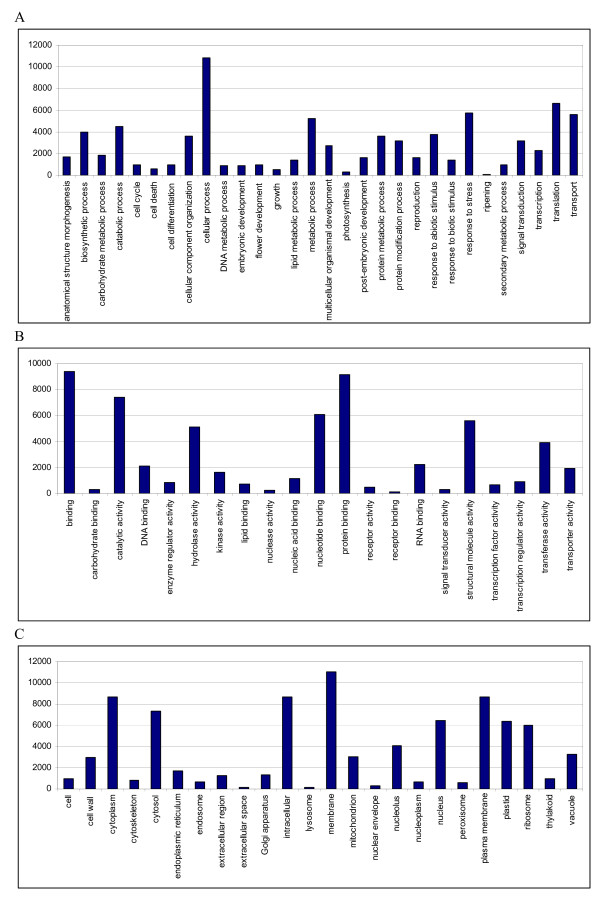
**Functional classification of watermelon unigenes within the category of biological process (A), molecular function (B) and cellular component (C)**.

Gene Ontology (GO) terms were then assigned to watermelon unigenes based on their sequence matches to known proteins in the UniProt databases and pfam domains they contain. A total of 33,853 unigenes (45.1%) were assigned with at least one GO term, among which 28,987 (38.6%) were assigned with at least one GO term in the biological process category, 28,997 (38.6%) in the molecular function category, and 27,036 (36%) in the cellular component category, while 21,779 (29%) unigenes were assigned GO terms in all three categories. Based on the GO annotations, watermelon unigenes were classified into different functional categories using a set of plant-specific GO slims, which are a list of high-level GO terms providing a broad overview of the ontology content. Cellular process, binding and membrane are the most abundant GO slims within the biological process, molecular function, and cellular component categories, respectively (Figure 4). Cellular process, metabolic process, and biosynthetic process were among the most highly represented groups within the biological process category, indicating the fruit flesh tissue was undergoing extensive metabolic activities. It is worth noting that GO annotations revealed a large number of expressed genes involved in carbohydrate metabolic process (1,807), anatomical structure morphogenesis (1,687), cellular amino acid and derivative metabolic process (1,595), and secondary metabolic process (992). In addition, genes involved in other important biological processes such as stress response, signal transduction, cell differentiation and fruit ripening were also identified.

To further identify active biochemical pathways during watermelon fruit development and ripening, we predicted biochemical pathways from our EST collection using the Pathway Tools [19]. A watermelon metabolic pathway database has been constructed and is freely available at the Cucurbit Genomics Database [16]. The database contained 318 metabolite pathways which were predicted from 2,968 enzyme-coding unigenes. Most major plant biochemical pathways such as calvin cycle, cellulose biosynthesis, ethylene biosynthesis, glycolysis II and IV, gluconeogenesis, and sucrose degradation, and several important secondary metabolite biosynthesis pathways including the citrulline-nitric oxide cycle, trans-lycopene biosynthesis, beta-carotene biosynthesis and flavonoid biosynthesis, were well covered by our EST collection.

### Watermelon simple sequence repeats (SSRs)

During the past several years, SSR markers have been extensively used in cucurbit species such as cucumber and melon for construction of high-density genetic maps and identification of QTLs associated with economically important traits [20-22]. However, due to the limited resources of watermelon sequences, SSR markers in watermelon have been scarce. We screened watermelon unigenes for the presence of di-, tri-, tetra-, penta- and hexa-nucleotide SSR motifs and were able to predict 5,195 SSRs from 4,668 watermelon unigenes, among which 2,265, 2,709, 115, 57, and 49 were di-, tri-, tetra-, penta- and hexa-nucleotide SSR motifs, respectively. The most frequent SSR motif was AG/CT (1,616; 31.1%), followed by AAG/CTT (1,300; 25%), AT/AT (519, 10%), and AAT/ATT (465; 9%). Primer pairs were designed for SSR motifs that had sufficient flanking sequences. The complete list of SSR motifs and their corresponding primer pair information are provided in Additional file 2.

The SSRs identified in the present study provide a rich resource of valuable molecular markers in watermelon. However, polymorphism of these SSRs will of course need to be tested in specific resource populations.

### Dynamic changes of gene expression and metabolite profiles during fruit development

Fruit development and ripening is a genetically programmed event that is defined by a series of biochemical and physiological changes that ultimately alter the fruit color, aroma, texture and its nutritional values. Researches toward elucidating the molecular basis of fruit development and ripening have been mainly performed on tomato, a typical climacteric fruit and model organism for fruit ripening, and numerous ripening-related genes have been isolated in tomato [15]. However, current available information regarding the physiology, biochemistry and molecular biology of fruit development and ripening of watermelon, a non-climacteric fruit, is very limited. To gain more insights into molecular mechanisms of watermelon fruit development and ripening, we performed comprehensive digital expression analysis using ESTs generated from the four stages described above.

Digital expression profiling (or RNA-seq) is a powerful and efficient approach for large-scale gene expression analysis [23]. Previous reports have described the identification of important genes through digital expression analysis with collections of relatively small number (1000 to 100,000) of tags [24,25]. In the present study, we have generated at least 125,000 tags for each of the four samples (Table 1), thus we should be able to capture the majority of moderately and highly expressed genes that are of interest. To validate the data from our digital expression analysis, qRT-PCR assays were performed on two sugar metabolism genes. The results showed that although the exact fold changes of selected genes at several data points varied between digital expression and qRT-PCT analyses, trends of gene expression changes detected by the two different approaches were largely consistent (Table 4). This confirmed the robustness of our digital expression data. In addition, previous reports indicated the increased expression of phytoene synthase 1 [3,26] and decreased expression of lycopene beta cyclase [26] during watermelon fruit development, which is consistent with our digital expression data (Table 5). This further validated our digital expression results.

**Table 4:** Validation of microarray results by qRT-PCR

Unigene	Method	10 DAP	18 DAP	26 DAP	34 DAP
WMU23179	Digital expression	0	6.8	19.2	68.1
	qRT-PCR	1	2.49	1.79	7.77
WMU23817	Digital expression	95.4	81	140.8	265.6
	qRT-PCR	1	7.45	1.81	7.7

Note: Digital expression data is shown as reads per million and qRT-PCR results were shown as relative fold changes compared to 10 DAP

**Table 5:** Carotenoid and sugar metabolism pathway genes that are differentially expressed during watermelon fruit development

Unigene	Description	Expression (reads per million)			
		**10 DAP**	**18 DAP**	**26 DAP**	**34 DAP**

WMU38667	phytoene synthase 1	0	81	76.8	47.7
WMU41454	lycopene beta cyclase	95.4	40.5	25.6	0
WMU23179	sucrose-phosphate synthase	0	6.8	19.2	68.1
WMU23817	sucrose synthase	95.4	81	140.8	265.6

Our digital expression analysis identified a total of 3,023 differentially expressed genes with at least two-fold difference in expression levels during watermelon fruit development and a false discovery rate (FDR) < 0.01 (Additional file 3). From this list of differentially expressed genes, we identified significantly enriched GO terms (FDR < 0.01), which serve as indicators of significantly altered biological processes during watermelon fruit development (Additional file 4). Among them are a number of interesting biological processes that are known to be associated with fruit development, including GO terms related to responses to different abiotic/biotic stresses, secondary metabolic process, organic acid metabolic process, and flavonoid biosynthetic/metabolic processes. We then further identified significantly altered biochemical pathways during watermelon fruit development; among which are several important pathways that are known to affect fruit quality, such as pathways of cellulose biosynthesis, suberin biosynthesis, sucrose biosynthesis and degradation, and starch biosynthesis and degradation (Additional file 5).

We further classified differentially expressed genes into different categories according to their expression patterns: 1) genes that are highly expressed in each of the four stages; 2) genes that are highly expressed in early stages; 3) genes that are highly expressed in late stages (Additional file 3). We also generated profiles of three major sugars (sucrose, fructose and glucose) that determine the sweetness of fruit and three carotenoids (lycopene, β-carotene and lutein) that play a critical role in fruit coloration and contribute significantly to fruit phyto-nutrient values. An integrative analysis of gene expression and metabolite profiles was performed to gain a better understanding of important fruit quality-related traits in watermelon.

#### Cell wall-related genes are highly expressed in immature white fruits

Immature watermelon fruits undergo a burst of cell division and later continue cell expansion to form large vacuolated cells that make up the majority of the flesh tissue [27]. Cell expansion involves changes in cell wall structure and continuous accumulation (in the vacuoles) of carbohydrates, organic acids, and different compounds needed to retain the osmotic pressure and flow of water into the expanding cells [28]. Through digital expression analysis, we identified a number of cell wall related genes that were expressed significantly higher in immature white fruits, including PRPs (proline-rich proteins), fasciclin-like arabinogalactan proteins (FLAs), xyloglucan endotransglycosylases (XETs) (Additional file 3). PRPs are a group of cell wall proteins characterized by their proline and hydroproline-rich repetitive peptides. They have been reported to be involved in cell wall formation and cell expansion in Arabidopsis, carrot and cotton [29-31]. FLAs are a subclass of arabinogalactan proteins (AGPs) that contain putative cell adhesion domains known as fasciclin domains. In eukaryotes, fasciclin domain-containing proteins are involved in cell adhesion and cell expansion [32-35]. During cell expansion and elongation, the cell wall continually undergoes temporary loosening followed by rapid reinforcement of wall structure. XETs are unique enzymes in plants that are capable of modulating the chemistry of the matrix and therefore performing both of these functions [36]. XETs have proved to catalyze the formation of covalent linkages between xyloglucans and cellulosic substrates and between xyloglucans and (1,3; 1,4)-β-D-glucans, and influence cell wall strength, flexibility and porosity, and cell expansion [36-38].

Several other genes involved in cell division and expansion were also identified as highly expressed genes in immature white fruits, including early nodulin-like proteins (ENODs), S-adenosyl methionine decarboxylase (SAMDC) and auxin-repressed protein ARP1. ENODs have been reported to function in cell-to-cell signaling, cell differentiation, tissue development, and signal transduction pathways [39,40]. SAMDC is the key gene involved in the biosynthesis pathway of polyamines (PAs), which are related to the cell growth and division in the early fruit development of many plant species [41-44]. Auxin is a plant growth hormone with many roles in cell division and enlargement, differentiation, and vascular bundle formation [45,46]. The expression of auxin responsive genes was also up-regulated in the rapid expansion phase of tomato fruit and in the fast fiber cell elongation stage of cotton [47-49]. These genes might play important roles in contributing to the fast growth of the early stage watermelon fruits.

#### Initiation of pigment biosynthesis in white-pink flesh fruits

**Figure 5:**
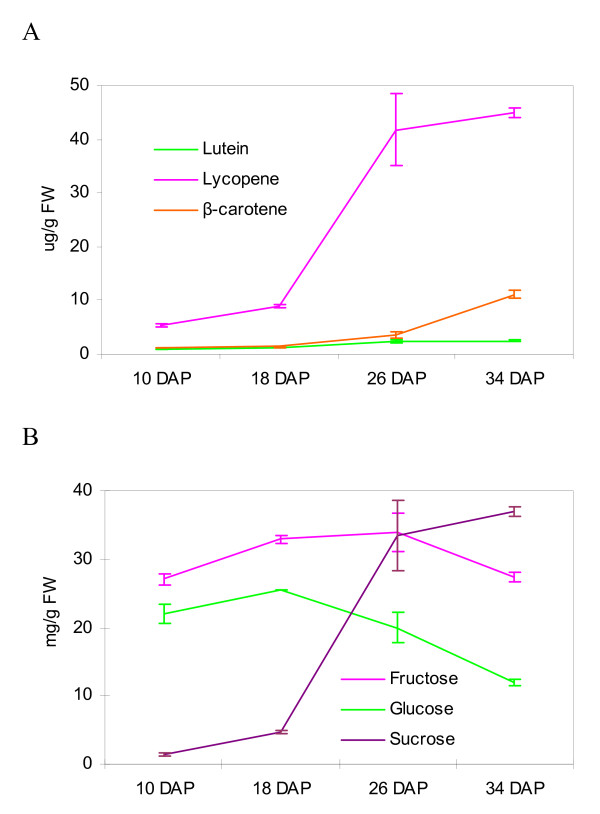
**Carotenoid (A) and sugar (B) content during watermelon fruit development**. The content of carotenoids including lycopene, β-carotene and lutein, and sugars including sucrose, glucose and fructose, was measured at four stages of fruit development: immature white (10 DAP), white-pink flesh (18 DAP), red flesh (26 DAP) and over-ripe (34 DAP). DAP: days after pollination. Each data represents mean ± SE (n = 2).

The color of watermelon flesh is an important quality trait and mainly determined by its carotenoid composition and content. Watermelon is a natural source of lycopene, a carotenoid that contributes the red color to watermelon flesh and is known for its antioxidant properties, acting as a potent free radical scavenger [50]. At the immature white stage, there is little difference between the inner peel and the flesh tissue in term of color for watermelon fruits, while at the white-pink flesh stage, its flesh tissue starts to turn pink due to the accumulation of lycopene (Figure 1). In the present study, we determined the content of three major carotenoids, lycopene, β-carotene and lutein, at the four stages of watermelon fruit development. As shown in Figure 5A, lycopene was the dominant carotenoid in watermelon fruit. Its content in immature fruits was low, and then increased slightly in white-pink flesh fruits but enough to make the visible pink color of the flesh (Figure 1). The lycopene content increased sharply in mature fruits (red flesh and over-ripe stages). The content of β-carotene kept at a very low level till the over-ripe stage, which had β-carotene content 3-5 times of previous stages. The content of lutein remained at the very low level throughout the watermelon fruit development (Figure 5A). The results we obtained for carotenoid content during watermelon fruit development are similar to those reported in tomato [51]. We then analyzed the expression of watermelon genes in the carotenoid biosynthesis pathway and identified two genes, a phytoene synthase 1 (WMU38667) and a lycopene beta cyclase (WMU41454), showing differential expression during fruit development (Table 5). The expression of the phytoene synthase 1 was not detectable at immature white stage and remained high since the white-pink flesh stage, while the expression of the lycopene beta cyclase kept decreasing during the fruit development and was undetectable at the over-ripe stage. This suggests that the up-regulation of the phytoene synthase 1 could have generated a flux of carotenoids and the down-regulation of lycopene beta-cyclase creates a blockade downstream, leading to the accumulation of lycopene. This is consistent with the findings in tomato [52]. Based on these results we concluded that phytoene synthase 1 and lycopene beta cyclase were key enzymes controlling carotenoid content in watermelon fruits and the regulatory mechanisms of carotenoid content during fruit development could be conserved between watermelon and tomato.

It is worth noting that a cinnamoyl-CoA reductase (CCR; WMU10737) was highly expressed in the fast-growing fruit (immature white and white-pink flesh). CCR catalyses the first step in the biosynthesis pathway of monolignols, the monomeric units of lignins. Lignified tissues play important roles in structural support and water/nutrient conduction [53]. Consistent with its role in lignin biosynthesis during plant development, expression of the CCR gene was shown to be high in tissues undergoing active lignification, i.e. vascular cambium and differentiating xylem. Development of vascular system in fleshy fruits is a critical component of fruit growth and expansion, because fruits serve as the nutrient sink and the vascular system provides the framework for water, nutrients, and sugars to be transported from the vegetative parts [3]. Thus, continued growth and expansion of watermelon fruits at early stages could rely on the establishment of the vascular system and similar connection between fruit development and vascular system formation has been reported in other fleshy fruit species like strawberry [54].

#### Rapid accumulation of sugars and secondary metabolites in mature fruits

Watermelon fruit at red flesh and over-ripe stages becomes much crispier and sweeter, and its flesh becomes red with accumulation of volatile compounds that gives watermelon its distinct aroma and flavor (Figure 1 and Additional file 1). Besides color, sweetness is another important trait of fruit quality and there has been enormous interest from growers and breeders to improve the sugar content of watermelon. Its sugar composition, as in many cucurbit fruits, is mainly determined by sucrose, fructose and glucose levels [5]. In the present study, we measured the content of these three sugars during watermelon fruit development. At immature white and white-pink flesh stages, fructose and glucose are the predominant sugars and their levels start to decline as the fruit matured. On the contrary, the content of sucrose was low in immature white and white-pink flesh fruits and then had a sharp increase in mature fruits, in which sucrose replaced fructose and glucose as the determining factor of sugar content (Figure 5B). Previous reports indicated that the sharp increase in sucrose level in a high-sucrose-accumulating watermelon cultivar was a result of fructose and glucose reduction, and sucrose made up about 70% of the total reducing sugars in mature fruit [5]. Our data confirmed the similar trend of sugar metabolism and, in the cultivar we examined, sucrose accounted for approximately 50% of total soluble sugar in over-ripe fruits, compared to 3-7% in fruits at immature and white-pink flesh stages (Figure 5B). We further analyzed sugar metabolism-related biochemical pathways and identified a sucrose synthase and a sucrose-phosphate synthase that were differentially expressed during watermelon fruit development (Table 5). These two genes were involved in the biosynthesis of sucrose and were highly expressed in fruits at red flesh and over-ripe stages. Correlation between sugar content and expression profiles of the two sugar metabolism related genes indicated that these two genes might play important roles in regulating sugar content in watermelon fruits.

A number of genes involved in the accumulation of second metabolites contributing to fruit flavor and aroma were found to be highly expressed in fruits at red flesh and/or over-ripe stages. They included ascorbate peroxidase (WMU23118), 9-cis-epoxycarotenoid dioxygenase (WMU05613), benzoquinone reductase (WMU23598), quinone reductase (WMU39499), flavonoid 3',5'-hdyroxylase (WMU39890), and squalene synthase (WMU27722 and WMU15567). Identification of these genes provided a rich source for further dissection of molecular mechanisms that governing fruit flavor and aroma, the important traits of fruit quality that currently are not well understood.

## Conclusion

Watermelon is an important fruit crop and becomes a useful model for the research of non-climacteric fruits. However, genetic and genomic resources for watermelon are very scarce, which are among the major limiting factors in watermelon research and breeding. In the present study, using the Roche/454 massively parallel pyrosequencing technology, we have generated more than half million watermelon ESTs from four stages of watermelon fruit development. These ESTs were *de novo *assembled and extensively annotated. They represent the significant expansion of the watermelon transcript catalog and provide a comprehensive material basis for future functional and expression analysis of genes of interest. The availability of these ESTs will also facilitate the annotation of the watermelon genome that is currently being sequenced. SSRs were also identified from these ESTs, which provides a valuable resource for the development of molecular markers that can be further used to facilitate the watermelon breeding program and cloning genes of interest. Integrative analysis of digital gene expression and metabolite profiles provided novel insights into molecular mechanisms of watermelon fruit development and regulatory mechanisms of several import traits of watermelon fruit quality.

## Methods

### Plant materials

Seeds of watermelon inbred line (*Citrullus lanatus *(Thunb.) Matsum. & Nakai var. *lanatus *cv 97103) were germinated and grown in greenhouse with nutrition pots containing a soil mixture (peat:sand:pumice, 1:1:1, v/v/v). Flowers were hand-pollinated and tagged. Center fruit flesh samples were collected at stages of 10, 18, 28, and 34 DAP, respectively. Tissues were immediately frozen in liquid nitrogen and stored at -80°C till use.

### cDNA preparation and sequencing

Total RNA was extracted from watermelon fruit flesh samples using the TRIzol Reagent (Invitrogen, USA). mRNA was purified from the total RNA using the Oligotex mRNA Midi Kit (QIAGEN, Germany). Double-strand cDNA was then synthesized using the SMART cDNA Library Construction kit (Clontech, USA) following the manufacturer's instructions. PCR products of cDNA were further purified using the QIAquick PCR Purification Kit (QIAGEN, Germany) and checked for quality using the Agilent 2100 Bioanalyzer. Approximately 10 ug cDNA from each of the four fruit flesh samples was used for sequencing on a Roche/454 GS-FLX Titanium instrument. A half-plate sequencing run was performed for each sample at the Cornell University Life Sciences Core Laboratories Center following manufacturer's instructions.

### cDNA sequence processing, assembly, annotation and comparative genomics analysis

The raw 454 sequence files in SFF format were base called using the Pyrobayes base caller [55]. In addition, around 12,000 watermelon ESTs were collected from GenBank. All these sequences were then processed to remove low quality regions and adaptor sequences using programs LUCY [56] and SeqClean [57]. The resulting sequences were then screened against the NCBI UniVec database, *E. coli *genome sequences, and watermelon ribosomal RNA sequences, to remove possible contaminations of these sequences. Sequences shorter than 100 bp were discarded. The processed 454 and GenBank sequences were assembled into unigenes using the iAssembler program [58].

The resulting watermelon unigene sequences were compared against GenBank non-redundant protein (nr) and UniProt (TrEMBL and SwissProt) databases using the BLAST program with a cutoff E-value of 1e-5. The unigene sequences were also translated into proteins using ESTScan [59] and the translated protein sequences were then compared to pfam domain databases. Gene ontology (GO; [60]) terms were assigned to each unigene based on the GO terms annotated to its corresponding homologues in the UniProt databases and domains in pfam database [61]. GO annotations of watermelon unigenes were then mapped to a list of plant-specific GO slim ontology [62]. Annotations of unigenes were then formatted into the PathoLogic format and used to predict watermelon biochemical pathways using the Pathway Tools [19].

For comparative genomics analysis, watermelon unigenes were compared to protein databases of cucumber (version 2), a closely-related cucurbit species, Arabidopsis (TAIR version 10), a model system of dicot plants, and rice (RGAP 6.1), a model system of monocot plants, using the BLAST program with an E-value cutoff of 1e-5. Cucumber, Arabidopsis and rice protein databases were obtained from the following links, respectively:

http://www.icugi.org/cgi-bin/ICuGI/genome/cucumber/download.cgi;

ftp://ftp.arabidopsis.org/home/tair/Sequences/blast_datasets/TAIR10_blastsets/TAIR10_pep_20101214;

ftp://ftp.plantbiology.msu.edu/pub/data/Eukaryotic_Projects/o_sativa/annotation_dbs/pseudomolecules/version_6.1/all.dir/all.pep

### Watermelon SSR identification

SSRs in watermelon unigene sequences were identified using the MISA program [63]. The minimum repeat number was six for dinucleotide and five for tri-, tetra-, penta- and hexa-nucleotide. Primer pairs flanking each SSR loci were designed using the Primer3 program [64].

### Identification of differentially expressed genes

Following cDNA sequence assembly, EST copy numbers of each unigene in each of the four stages of fruits were derived and used as an approximate estimation of gene expression levels in the corresponding tissues following normalization to RPM (reads per million sequenced reads). Significance of differential gene expression during watermelon fruit development was determined using the R statistic [65] and the resulting raw p values were corrected for multiple tests using the False Discovery Rate (FDR; [66]). Genes with expression changes no less than two during fruit development and FDRs less than 0.01 were identified as differentially expressed genes. GO terms enriched in the set of differentially expressed genes were identified using GO::TermFinder [67], requiring p values adjusted for multiple testing to be less than 0.01. Significantly altered pathways during fruit development were identified using the Plant MetGenMAP system [68].

### Determination of the content of sugars, soluble solids and carotenoids, and flesh firmness

Sugars including sucrose, glucose and fructose were determined according to protocols described in Bethke et al. [69] and Yativ et al. [5], with minor modifications. Two hundred milligrams of frozen watermelon flesh samples were ground to a fine powder prior to being extracted for 1 h in 10 ml of 50% ethanol at 80°C, then centrifuged at 3000 g for 10 min. This step was repeated one more time and the supernatants were then dissolved to a volumetric flask (25 ml) as extracts. Two milliliter extracts were centrifuged at 3000 g for 10 min. The supernatants (1 ml) were filtered through a 0.45-mm HPLC nylon filter (Membrana, Germany). Sugars were separated in an analytical HPLC system (Pump System LC-10ATVP, Shimadzu, Japan) fitted with a Shodex Asahipak NH2P-50 4E column (4.6 × 250 mm, Shodex, Japan) using a refractive-index detector (RID-10A, Shimadzu, Japan). Soluble solid content (SSC) was measured using juices extracted from the center flesh of watermelon fruits with a digital refractometer (Digital Refractometer PR-1, Atago, Japan). The content of three carotenoids (lycopene, β-carotene and lutein) was determined according to the protocol described in Fraser et al. [51]. Individual carotenoids were separated by HPLC on a Waters Nova-pak C18 column. Flesh firmness was measured on the center of watermelon flesh, using a pressure tester (FT327, Breuzzi, Italy). All the above experiments were performed using two biological replicates.

### qRT-PCR analysis

Two sugar metabolism genes (WMU23179, sucrose-phosphate synthase and WMU23817, sucrose synthase) were selected for qRT-PCR analysis. qRT-PCR reactions were run in a Roche 480 Real-Time PCR system. Each 20 μL reaction consisted of 5 μL cDNA (~20 ng/uL), 5 μL of primer mix (2 μM of each forward and reverse primer), 10 μL of lightcycler 480 SYBR Green I Master Mix. Cycling conditions were 95°C for 3 min, followed by 45 cycles of: 95°C for 10 sec, 58°C for 20 sec, and 72°C for 30 sec. Melting curves were performed at the end of each reaction run to detect primer dimers. The 18S rRNA gene was used as the internal control. Quantification was achieved by normalizing the number of target gene copies to a reference 18S rRNA gene by using the comparative Ct method [70]. The ΔCt was calculated by subtracting the average Ct value of each tissue type from the average Ct values of 18S rRNA. The ΔΔCt was calculated by subtracting the ΔCt of each of the four fruit stages from the ΔCt of the fruit tissue. The formula 2 ^-(ΔΔCt) was used to calculate a relative fold change between the four fruit developing stages. Primer pairs used for WMU23179, WMU23817, and 18S rRNA were F:5'-TAACCTAGTGGTTGTGGCTGGAGA-3' and R:5'- TGGCCATTCAGGTTGTAGGTCT-3', F:5'-TCGCATCAAGAGCTCAAGCACTCA-3' and R:5'- GCACTTGCATCACCTGGTTTCCAT-3', and F:5'-AGCCTGAGAAACGGCTACCACATC-3' and R:5'-ACCAGACTCGAAGAGCCCGGTAT, respectively.

## Authors' contributions

SG, YZ and MH performed data analysis. HH and JL measured metabolite contents. HZ and YR prepared cDNA samples for 454 sequencing. GG performed the field planting and management. SZ helped in data interpretation and manuscript writing. ZF and YX designed the experiment and provided guidance on the whole study. ZF was also involved in sequence analysis and wrote the manuscript. All authors have read and approved the manuscript.

## Supplementary Material

Additional file 1 **Changes of soluble solid content (SSC), flesh firmness, and fruit weight during watermelon fruit development**. Click here for file

Additional file 2 **watermelon SSRs**. The table provides the list of SSRs identified from watermelon ESTs, their motif sequences and surrounding primer pair information Click here for file

Additional file 3 **Differentially expressed genes**. The table provides the list of differentially expressed genes during watermelon fruit development Click here for file

Additional file 4 **Enriched Gene Ontology (GO) terms**. The table provides the list of enriched GO terms within the biological process category identified from differentially expressed genes during watermelon fruit development Click here for file

Additional file 5 **Significantly altered pathways**. The table provides the list of significantly altered biochemical pathways during watermelon fruit development Click here for file
