# Supplementary material for: Characterization of transcriptome dynamics during watermelon fruit development: sequencing, assembly, annotation and gene expression profiles
Source: BMC Genomics. 2011 Sep 21;12:454. doi: 10.1186/1471-2164-12-454 (PMC3197533; doi:10.1186/1471-2164-12-454)
Supplement: Additional file 1 — Changes of soluble solid content (SSC), flesh firmness, and fruit weight during watermelon fruit development. [file 1471-2164-12-454-S1.PDF]

A

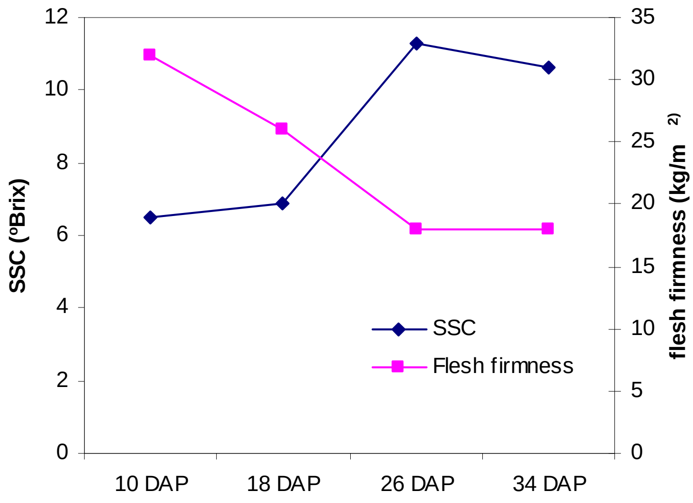

B

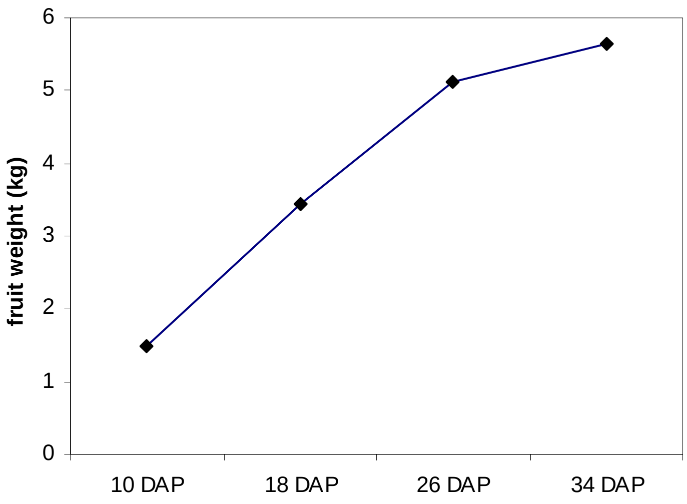

Changes of soluble solid content (SSC; left axis) and flesh firmness (right axis) (A) and fruit weight (B) during watermelon fruit development.

DAP: days after pollination
